# CARs: Beyond T Cells and T Cell-Derived Signaling Domains

**DOI:** 10.3390/ijms21103525

**Published:** 2020-05-15

**Authors:** Nico M. Sievers, Jan Dörrie, Niels Schaft

**Affiliations:** 1 Department of Dermatology, Universtitätsklinikum Erlangen, Friedrich-Alexander-Universität Erlangen-Nürnberg, Hartmannstraße 14, 91052 Erlangen, Germany; nico.sievers@extern.uk-erlangen.de (N.M.S.); Jan.doerrie@uk-erlagnen.de (J.D.); 2 Comprehensive Cancer Center Erlangen European Metropolitan Area of Nuremberg (CCC ER-EMN), Östliche Stadtmauerstraße 30, 91054 Erlangen, Germany; 3 Deutsches Zentrum Immuntherapie (DZI), Ulmenweg 18, 91054 Erlangen, Germany

**Keywords:** chimeric antigen receptor (CAR), intracellular signaling domain, T cell, NK cell, NKT cell, γ/δ T cells, myeloid cells, NKG2D, DAP10, 2B4

## Abstract

When optimizing chimeric antigen receptor (CAR) therapy in terms of efficacy, safety, and broadening its application to new malignancies, there are two main clusters of topics to be addressed: the CAR design and the choice of transfected cells. The former focuses on the CAR construct itself. The utilized transmembrane and intracellular domains determine the signaling pathways induced by antigen binding and thereby the cell-specific effector functions triggered. The main part of this review summarizes our understanding of common signaling domains employed in CARs, their interactions among another, and their effects on different cell types. It will, moreover, highlight several less common extracellular and intracellular domains that might permit unique new opportunities. Different antibody-based extracellular antigen-binding domains have been pursued and optimized to strike a balance between specificity, affinity, and toxicity, but these have been reviewed elsewhere. The second cluster of topics is about the cellular vessels expressing the CAR. It is essential to understand the specific attributes of each cell type influencing anti-tumor efficacy, persistence, and safety, and how CAR cells crosstalk with each other and bystander cells. The first part of this review focuses on the progress achieved in adopting different leukocytes for CAR therapy.

## 1. Conventional T Cells Are the Pioneers of Chimeric Antigen Receptor (CAR) Therapy

T cells are characterized by the possession of a T cell receptor (TCR), in most T cells, consisting of the α and β TCR chains. Mature T cells divide into cell fates defined by the surface co-receptor molecules CD8 (cytotoxic T lymphocytes) and CD4 (T helper and regulatory T cells). Independently of CD4 and CD8, T cells can differentiate from a naïve state (T_N_) towards an effector (T_E_) or a memory (T_M_) phenotype, which is further subdivided in the central memory (T_CM_) and the effector memory (T_EM_) compartment, which differ in their self-renewal capacity and effector functions [1,2,3,4,5,6,7].

**Figure 1:**
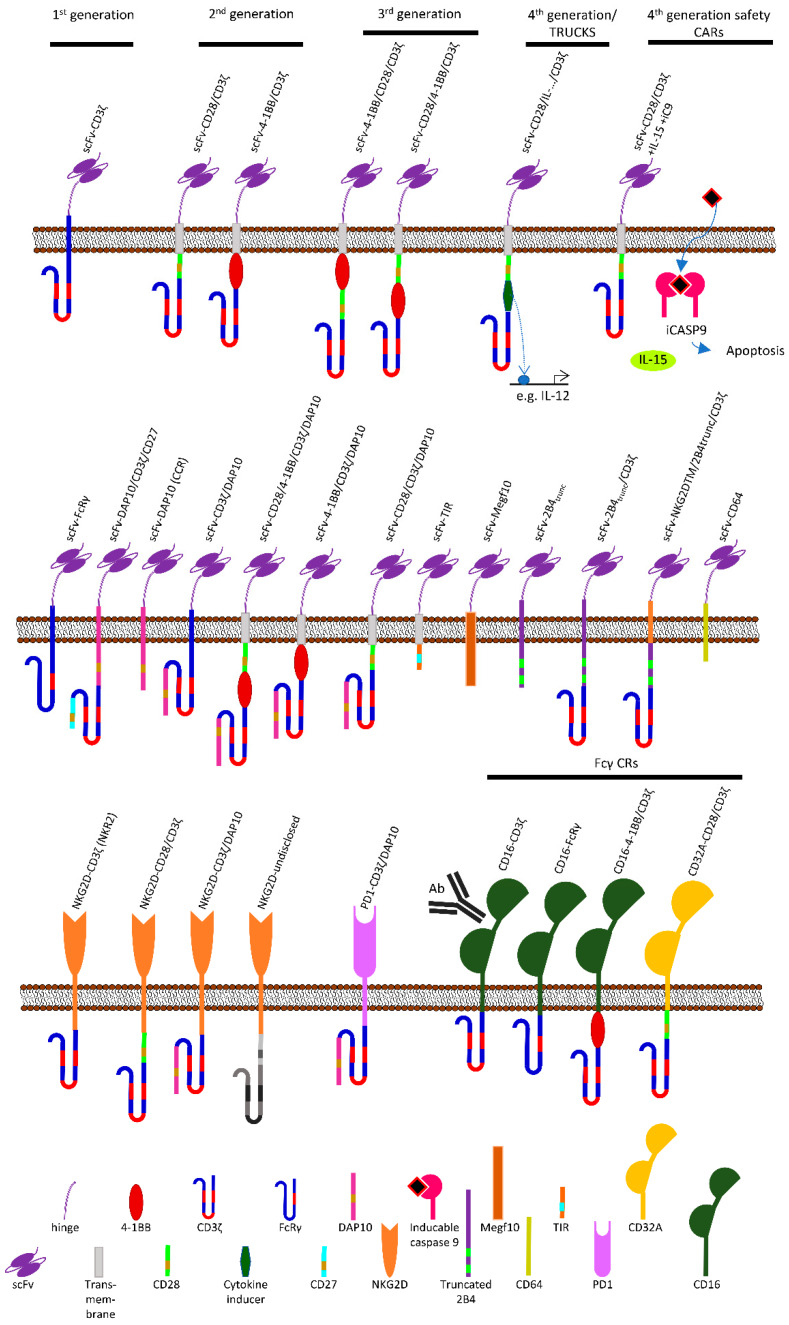
Schematic representation of all the CARs described in this review. Upper membrane: classical CAR models, lower two membranes: the more exotic CAR models.

T cells are clearly the frontrunners of CAR therapy. The first ever CAR created by Gross et al., named T body at that time, was an anti-CD19-CD3ζ CAR (Figure 1) retrovirally transduced into peripheral blood T cells [8]. Over the years, T cells always stayed in the focus of research, with most CAR constructs being designed specifically for this cell type. The greatest success in the CAR field so far and a milestone in cellular therapy was achieved when two autologous anti-CD19-CAR T cell therapies against B cell lymphoma (Kymriah^®^ (Tisagenlecleucel) and Yescarta^®^ (axicabtagen-ciloleucel)) were approved by the Food and Drug Administration (FDA) [9], reaching an astonishing remission rate of 80% [10].

When talking about T cells as CAR vehicles in a generalized way, we must keep in mind that different subpopulations exist. Many published reports did not further differentiate the subtypes and lineages within the expanded T cell pool, meaning that an unknown composition of CD4^+^, CD8^+^, naïve, effector, and memory T cells was administered [7]. This becomes important knowing that the frequency of these subsets can differ markedly in individuals because of factors such as age, pathogen exposure, or lymphocytotoxic medications [11,12]. The heterogeneity of T cell subsets may have influenced efficacy and toxicity in clinical trials and could explain part of the variations observed [13,14,15,16], as there are several studies pointing out the influence of the subset distribution on anti-tumor response and persistence [7,17,18,19]. While CD8^+^ T_EM_ and T_CM_ cells yield the best in vivo persistence of all subsets [20,21], T_N_ and T_CM_ show stronger anti-tumor activity than T_EM_ cells [22,23]. Unfortunately, the T_EM_ subset is usually increased in cancer patients compared to healthy controls [7]. All CD4^+^ subsets have less cytolytic potential, but show stronger cytokine secretion than CD8^+^ cells, matching their native role during an immune response [7]. Among both CD4^+^ and CD8^+^ T cells, cytokine production is higher in T_N_ than in further differentiated compartments [7]. Sommermeyer et al. determined an ideal cell cocktail to contain 1:1 CD8^+^ CAR-T_CM_ to CD4^+^ CAR-T_N_ cells in a mouse model of Raji lymphoma [7], suggesting that IL-2 produced by CD4^+^ cells drives optimal proliferation of CD8^+^ CAR-T cells, which are then the main component of anti-tumor cytotoxicity [7,19,24,25]. These findings have been successfully translated to a phase 1/2 clinical trial of an anti-CD19 CAR against acute lymphoblastic leukemia (ALL) (NCT01865617) [26].

Although undoubtedly conventional α/β T cells are the biggest players in the field of CAR cell therapy in the clinics, there are many more cellular vessels to be considered. We will summarize findings with these cell types below.

## 2. Alternative Cell Types Suitable for CAR Cell Therapy

While having proven their potential in the treatment of hematological cancers [27,28,29,30], CAR therapies have not yet been successfully translated to solid cancers [31,32]. One main hurdle here is the immunosuppressive tumor microenvironment (TME) that impairs recruitment of effector cells and drives them into anergy [33]. Intensifying the treatment, e.g., by increasing the administered dose or by generating more potent CAR effector cells to enhance anti-tumor efficacy, often brings along severe side effects such as cytokine release syndrome (CRS), on-target/off-tumor toxicity, or neurotoxicity [15,16,19,34,35]. This demands for a balance to be found between efficacy and toxicity.

Although α/β T cells remain a very promising vehicle for future CAR therapies, research efforts on other cell types to be used alternatively must be continued. The intrinsic features and unique anti-tumor capabilities of different immune cells might prove better suitable to overcome current hurdles in both efficacy and safety of CAR therapy. This includes abilities to evade suppression by the TME, to drive a broader immune response thanks to cross-interactions among different cell types, to not be restricted to the autologous setting (as conventional α/β T cells are prone to induce graft versus host disease (GvHD), and the reduced tendency towards autoimmunity [36]. Properties and clinical data on the use of less common T cell subsets, NK cells, and myeloid cells as CAR vehicles are summarized in the following.

### 2.1. γ/δ T Cells

Besides conventional α/β T cells, γ/δ T cells came into focus as potential vehicles for CAR therapy. Their endogenous T cell receptor (although undergoing gene rearrangement) possesses decreased variability and is not restricted to antigens presented on major histocompatibility complex (MHC) [37]. Only some antigens recognized by γ/δ TCRs have been determined so far, e.g., some uncommon nucleotides, phospholipids, and heat shock proteins (HSPs). These are not pathogen-specific themselves but tend to appear on infected or malignant cells under stress, showing the ability of γ/δ TCRs to act as pattern recognition receptors (PPRs) [38]. Additionally, some γ/δ TCRs were shown to bind “non-classical” MHC-Ib molecules and peptides presented on these. Uniting aspects of innate and adoptive immunity, γ/δ T cells are thought to play a “transitional”, “in between” role in the immune system [39,40]. γ/δ T cells are especially prominent in the defense of epithelial tissue, such as the skin or the gut, as part of the population of intraepithelial lymphocytes (IELs) [39]. IEL γ/δ T cells predominantly carry a TCR Vδ1 chain and make up the largest quantity of γ/δ T cells, while a Vδ2 chain carrying subpopulation is circulating in the blood, representing up to 10% of all circulating T lymphocytes [37,41].

γ/δ T cells present within the tumor microenvironment were found to be a favorable diagnostic marker, hinting at these cells’ potential against aberrant cells [37,42]. On the other hand, γ/δ T cells exhibit certain immune regulatory functions in their role as tissue resident cells by secreting the anti-inflammatory cytokines IL-4, IL-10, and TGFβ, which impair effector cells, e.g., within the TME [42]. IL-17, secreted by γ/δ T cells, was even suggested to act tumorigenic by enhancing angiogenesis and the recruitment of macrophages and suppressive myeloid cells [43,44,45,46].

Because they lack affinity for MHC, γ/δ T cells do not exhibit alloreactivity, resulting in several advantages for the use in CAR therapy. γ/δ T cells can be prepared as an ‘off-the-shelf’ therapy when cultured from healthy donor cells [47]. While the patient’s leucocytes might be negatively impacted by previous chemotherapies, donor cells better exhibit their full potential [48]. Furthermore, γ/δ T cells are potentially better suitable to avoid tumor antigen evasion: besides the introduced CAR, they possess a strong anti-tumor potency through their native TCR and various other receptors, including the ability to lyse cells by antibody-dependent cellular cytotoxicity (ADCC) [37]. Blood circulating γ/δ T cells were shown to, under certain conditions, act as antigen-presenting cells (APCs) stimulating conventional α/β T cells [49]. These then launch a poly-antigen targeting attack on the tumor incorporating diverse α/β TCRs [50]. This also allows the buildup of anti-tumor memory, which is key towards long-term tumor control.

#### Clinical Trials Employing CAR-γ/δ T Cells

Until now, there are three studies listed on clinicaltrials.gov (Table 1), one assessing feasibility of γ/δ T cell isolation and expansion in preparation to testing an anti-CD33-CD28 CAR (Figure 1) (NCT03885076). The other two employ an anti-CD19 CAR against relapsed hematological malignancies (NCT02656147) respectively, an anti-NKG2DL CAR (Figure 1) against a variety of solid tumors (NCT04107142). Neither of them has shared details on their CAR constructs and no results have been published yet.

### 2.2. NKT Cells

Being less abundant in the blood than γ/δ T cells, NKT cells represent only around 0.1% of circulating T lymphocytes [51]. They co-express NK cell markers and a (semi) invariant α/β TCR, which is restricted to lipid antigens presented on CD1d [51,52]. Proficiency in FasL- and perforin-mediated killing, as well as the cytokine secretion profile, differ between subpopulations defined by CD4, CD8, and CD62L expression [53]. Just like γ/δ T cells, NKT cell invasion in a tumor is considered a favorable predictor for survival in colorectal carcinoma [54], but certain NKT cell subpopulations also have tumor-protective properties preventing immune surveillance [55]. NKT cells possess the ability to recognize and lyse tumor-associated macrophages (TAMs) via their TCR binding CD1d [56], improving tumor invasion and clearing the path for other cell types to follow up. Via CD40L, NKT cells promote the maturation of dendritic cells [57] and thereby the recruiting of α/β T cells to the TME, supporting antigen spread.

#### Clinical Trials Employing CAR-NKT Cells

An ongoing clinical trial by Heczey and colleagues (NCT03294954, replacing the withdrawn NCT02439788) is testing an (14G2a-mAB-derived) anti-GD2-CD28/IL-15 CAR (Figure 1) construct in autologous NKT cells (called GINAKIT2) (Table 1). Pre-clinical data showed that CD28 co-stimulation and co-expression of IL-15 yielded the best CAR cell durability, relocation to tumor sites, and tumor control in murine neuroblastoma and lymphoma models, while inducing no symptoms of GvHD. Alternatively, 4-1BB co-stimulation (Figure 1) in direct comparison led to reduced proliferation and increased activation-induced cell death.

A second ongoing clinical trial (NCT03774654) also initiated by the Baylor College of Medicine (TX, USA) uses an anti-CD19-CD28/IL-15 CAR construct in allogenic NKT cells against relapsed or refractory B-cell malignancies. The healthy donor-derived transduced CAR-NKT cells (called ANCHOR cells) are administered following immunodepleting therapy. Although previous clinical trials investigated the potential and safety of un-transduced autologous NKT cells in tumor treatment (NCT02619058, NCT01801852), the described two trials represent the first usage of CAR-NKT cells in humans as well as the first clinical use of allogenic NKT cells. To date, no results have been published.

### 2.3. NK Cells

Natural killer cells exhibit a strong endogenous anti-tumor capacity via a broad variety of natural cytotoxicity receptors (NCRs), stress receptors, and antibody binding Fc(γ) receptors [58], while their cytotoxicity is controlled via inhibitory stimuli from MHC-I binding receptors (e.g., Killer cell immunoglobulin-like receptors, KIRs). NK cells are constantly integrating these activatory and inhibitory signals and enter an activated state once this balance is shifted: upon encountering a cell having downregulated MHC-I (“missing-self”), upon receiving more activatory stimuli from a cell with aberrant surface markers or marked by antibodies (“induced-self”), or a combination of both [58,59]. To lyse tumor cells, NK cells are capable of cellular cytotoxicity and ADCC, as well as secreting cytokines promoting other leucocytes [60]. Two major subpopulations of NK cells have been defined: those residing predominantly in lymphoid organs are CD56^hi^/CD16^low^ and more proficient in cytokine secretion than in killing [59], while blood circulating NK cells make up around 6% of circulating lymphocytes, are CD56^low^/CD16^hi^, and have a higher cytolytic capacity [59].

The lack of alloreactivity allows the usage of heterologous NK cells for adoptive cellular therapy. Until now, most CAR-NK cell studies have stuck to the haplo-identical transplantation setting, although KIR:MHC-I mismatch between graft and recipient was demonstrated to enhance cancer control [61,62]. Clinical scale numbers of heterologous NK cells can be obtained in various ways: by cultivation from PBMCs using autologous PBMCs as feeder cells [63] or IL-15 and 4-1BBL expressing K562 feeders [64], or without any feeder cells [65]. Better persistence of CAR-NK cells was achieved when the cells had been isolated from cord blood [66] or generated by differentiation from gene-modified hematopoietic stem cells [67]. The use of induced pluripotent stem cells has also been described [68]. The NK-92 cell line allows simple and unlimited in vitro expansion of NK cells requiring only IL-2 to grow [69]. Although very convenient to use, this cell line was shown to be limited in its KIR repertoire and lacks CD16 expression, which might render it less effective in tumor control compared to primary NK cells [70]. Furthermore, NK-92 cells are aneuploid and must be irradiated before being administered, limiting their persistence in vivo [70].

#### Clinical Trials Employing CAR-NK Cells

Besides T cells, NK cells have received the second most attention in CAR research and have been featured in several clinical trials as a potent and save alternative with good pre-sets for future therapeutic application, as defined by pre-clinical data [71,72,73]. Currently (April 2020), a total of seventeen clinical trials employing CAR-transfected NK cells have been registered on clinicaltrials.gov (Table 2).

From all of these, only Liu et al. (NCT03056339) have published results by now [74]. In this phase 1/2 study, heterologous cord blood-derived NK cells have been retrovirally transduced with an anti-CD19-CD28/CD3ζ CAR transcriptionally coupled to IL-15, plus a safety switch in the form of inducible caspase 9 (Figure 1). They were employed against non-Hodgkin’s lymphoma and chronic lymphocytic leukemia (CLL) and achieved a response in eight out of eleven patients, with seven having complete remission. None of the patients showed critically increased levels of inflammatory cytokines or cytokine release syndrome, signs of neurotoxicity, or GvHD. Persistence of the CAR-NK cells was at least twelve months [74]. Of the other ongoing clinical trials, one addresses an anti-BCMA and one an anti-CD3 CAR, and all other trials target CD19. Those who have shared details on their CAR constructs are working on either 4-1BB/CD3ζ or CD28/4-1BB/CD3ζ CARs (Figure 1).

### 2.4. Myeloid Cells

Beside erythrocytes and platelets, the common myeloid progenitor gives rise to those cells forming the backbone of innate immunity: granulocytes, namely neutrophils, eosinophils, and basophils and mast cells, as well as monocytes, macrophages, and myeloid dendritic cells. Taken together, these cells make up more than half of all blood circulating leukocytes and carry out diverse roles in innate immunity, but also facilitate adaptive immunity.

Very little data on myeloid cells as CAR vehicles has been obtained so far [75,76,77,78] and no clinical trial is registered on clinicaltrials.gov. Other than lymphocytes, the rather short-lived mature granulocytes are difficult to expand in vitro [75]. To obtain therapeutically relevant numbers of CAR-myeloid cells, hematopoietic stem cells (HSCs) or myeloid progenitors can be transduced, (selectively) expanded, and grafted [75]. As a proof-of-principle, De Oliveira et al. retrovirally transduced cord blood-derived human CD34^+^ hematopoietic stem cells with CD19-specific CAR constructs [75]. After differentiation, the CARs were detectable on mature granulocytes, monocytes, and macrophages without impairing these cells’ conventional effector functions, plus granting them the ability to lyse CD19^+^ targets in vitro [75]. After transplantation, CAR-expressing neutrophils are expected to appear after about two weeks [36]. The gene-modified HSCs continuously gave rise to new CAR effector cells, including lymphocytes and myeloid cells, resulting in de facto infinite persistence of CAR cells in vivo [76]. While this solves the issue of cell exhaustion faced in conventional CAR-T cell therapy, it also calls for reliable safety switches to control toxicity and the risk of malignant transformation [76]. An example for a safety switch gene successfully tested by Kao et al. is the truncated epidermal growth factor receptor (EGFRt), mediating ADCC [79]. The presence of many different CAR effector cell types at the same time promises multi-level anti-tumor activity, employing all arms of the immune system [77]. Analogously, Hege et al. grafted HSCs transduced with a CD4/CD3ζ fusion construct into mice and measured cytotoxicity against HIV-1 gp120 targets [80]. To demonstrate the contribution of non-T cells to the cytotoxicity, they repeated the experiment in SCID mice (that are incapable generating mature T cells) [80]. Several other groups also proved HSC-derived CAR myeloid cells to significantly contribute to cytotoxicity of this multi-lineage approach [79,81,82]. Of note, most studies working on CAR-transduced HSCs do not address myeloid cells, but focus more on the potential of a self-renewing CAR-T cell response [78].

#### 2.4.1. Neutrophils

Neutrophils are not only the most abundant type of granulocytes but also the most abundant of all leukocytes, making up about 60% to 70% of them [83]. They are very short-lived, having a half-life of only about 18 h in vitro [81]. In an immune response, neutrophils pursue the role of a first line of defense by rapid infiltration of the affected tissue [83]. Only one study, by Roberts et al., has specifically looked at CAR neutrophils so far. The group transduced human HSCs with an anti-CD4-CD3ζ CAR and isolated CAR-expressing neutrophils to successfully test their cytotoxicity against Raji cells in vitro [81].

#### 2.4.2. Monocytes

Monocytes are large-sized leukocytes. Themselves acting as phagocytes in the innate immune system, they can give rise to macrophages and myeloid dendritic cells [84]. Unlike all previously mentioned studies on CAR-myeloid cells, Biglari et al. did not employ gene-modified HSCs but directly isolated peripheral blood human monocytes. They adenovirally transduced the monocytes with an anti-CEA-CD64 CAR (Figure 1) and documented reduced tumor progression in treated mice, accompanied by strong TNF secretion [85].

#### 2.4.3. Macrophages

Macrophages are the most prominent type of phagocyte in the immune system and form several subpopulations with specialized roles. While M1 macrophages are effector cells of pathogen and tumor defense, M2 cells exhibit regulatory and tissue restauration abilities: resolving inflammation and wound healing (M2a), immunoregulation (M2b), immunosuppression and tissue remodeling (M2c), angiogenesis, and also tumor growth (M2d) [86]. This subdivision is not mutually exclusive and mixed phenotypes exist [87]. Macrophages are capable of killing target cells via phagocytosis and trogocytosis (the uptake of severed cell parts) [88,89,90] and MHC-II presentation of antigens taken up, as well as secretion of cytokine and effector molecules [91,92].

Macrophages possess the ability to infiltrate tumors in large numbers and are then referred to as tumor-associated macrophages (TAMs) [93], which can make up 50% of tumor mass [94]. Once in the TME, they lose their capability to migrate and to lyse tumor cells. Adopting an immunoregulatory phenotype, they even contribute to the suppressive TME and tumor growth [33,93,95]. Consequently, the presence of high numbers of TAMs is associated with poor clinical prognosis [93]. On the other hand, increasing the rate of macrophage phagocytosis (e.g., via CD47 blockage) boosts CD8^+^ T cell responses [96], as the presentation of antigens to T cells is a determining factor of the T cell anti-tumor response [96,97].

The high prevalence of macrophages in solid tumors makes them a promising vehicle for CAR therapy, provided that the suppressive influence of the TME can be overcome [89]. CAR macrophages could contribute to shifting the TME towards tumor rejection by releasing inflammatory cytokines such as TNF, IL-6, IFNγ, and IL-12 [89,98,99], by promoting T cell recruitment and function, and tumor growth suppression [99]. Tumor cell lysis was achieved via CAR-directed phago- and trogo-cytosis [71]. By presenting processed tumor antigens to T cells, CAR macrophages contributed to a poly-antigenic T cell response, reducing the risk of antigen evasion [89,90]. A down-point of macrophages as CAR vehicles is their endogenous resistance to viral infection, resulting in low transduction rates [100]. Adenoviruses yielded the best transduction efficacy but are associated with high immunogenicity and an inflammatory phenotype in target cells, which is a risk factor for clinical applications [89,98,100]. Electroporation was also shown to be inefficient and resulted in low viability [100].

Morrissey et al. lentivirally transduced murine bone marrow-derived macrophages (BMDMs) with anti-CD19 or anti-CD22 CARs using (amongst others) FcRɣ and Megf10 signaling domains (Figure 1) [90]. These so-called chimeric antigen receptors for phagocytosis (CAR-Ps) enabled macrophages to engulf differently sized bead targets and limit Raji cell growth in vitro [90]. Klichinsky et al. employed the THP-1 macrophage model cell line to express anti-CD19-, anti-meso-, and anti-HER2-CD3ζ CARs (CAR macrophages, CARMA) and showed them to selectively clear cognate antigen-bearing tumor cells, promote CTL proliferation, as well as to reduce tumor burden in an ovarian cancer xenograft model [89]. CARMA cells differentiated towards a durable M1 phenotype and resisted subversion to M2. The group also established a chimeric adenoviral vector, Ad5f35, that achieved around 70% transduction efficacy in macrophages [89].

Another group worked on macrophage Toll-like receptor-chimeric antigen receptors (MOTO-CARs), which have a Toll Interleukin receptor (TIR) signaling domain (Figure 1) [98]. Transduced into monocyte-derived human macrophages using Ad5f35, these receptors triggered phagocytosis of non-small cell lung carcinoma cell lines [98].

#### 2.4.4. Myeloid Dendritic Cells

Myeloid dendritic cells (DCs) are antigen-presenting cells (APCs) proficient in antigen uptake, processing, and presentation via MHC molecules. They serve as ambassadors between innate and adaptive immunity, being crucial for antigen-specific T cell responses. While no trial on CAR-transfected dendritic cells has been published so far, cellular vaccination strategies employing DCs are on the rise. Given that a suitable antigen is available, DCs modified to present this particular antigen can drive T cells against virus-infected or cancer cells and initiate multi-lineage immune responses through further cell–cell communications [101]. Immune memory against the designated target is also induced und can grant long-term immunity [101].

As DC vaccination (DCvac) and CAR-T cells are the two fastest growing arms of adoptive cell therapy, recent trials seek to combine these two in the siege against cancer. Wu et al. tested co-cultures of anti-CD19 CAR-T cells with EGFR pathway substrate 8 (Eps8)-presenting DCs in vitro, which enhanced proliferation and reduced AICD of T cells. A shift from the T_EM_ to the favorable T_CM_ compartment increased cytokine secretion (especially IL-2), and lytic capacity was reported when confronted with CD19^+^/Esp8^high^ leukemia cells [102]. Two clinical trials are registered approaching similar combination therapies (see Table 1). One (NCT03291444) combines a variety of CAR-T cells with Eps8 or WT1 peptide-loaded DCs against ALL, acute myeloid leukemia (AML), and myelodysplastic syndrome. The other (NCT04085159) targets neurofibromatosis with a triple-combination of CAR-T cells, DCvac, and engineered immune effector cytotoxic T cells, without granting more insight on their cells and constructs.

Although not redirecting them directly against target cells, Squadrito et al. presented DCs expressing a chimeric receptor increasing the acquisition of tumor antigens from cancer cell-derived extracellular vesicles (EVs) [103]. Their construct consists of an anti-HER2 single-chain variable fragment (scFv) coupled to a signaling-incompetent truncated nerve growth factor receptor, allowing the DCs to bind EVs carrying MHC-I:HER2-peptide complexes on their surface. Increased internalization of the EVs allowed cross-dressing with the antigen and activation of T cells in vitro [103].

Taken together, since the clinical efficacy of CAR-T cells against solid tumors lags behind their results against hematologic cancers, it is obvious that new approaches are necessary to overcome these limitations. Changing the cellular vehicle encompassing the CAR is one possible way addressed by more and more groups. The strategies listed above will need to prove their efficacy in clinical trials, some of which are currently running. Employing alternative cellular vehicles also demands for specifically optimized CAR constructs, which will be addressed below.

## 3. Alternative Extracellular and Intracellular Domains Used in CARs

The composition of chimeric antigen receptor constructs has been successively optimized since the first ever CAR was designed in 1989 [8]. The most common CAR design achieves recognition of a target antigen via a monoclonal antibody-derived single chain variable fragment (scFv) [104]. This extracellular domain is connected to intracellular signaling domains via a linker and a transmembrane (TM) region. In the simplest constructs, this signaling domain consists only of the CD3ζ chain from the TCR complex (Figure 1) [8]. Later improvements, referred to as second-generation CARs, added a second signaling domain chosen from the pool of known co-stimulatory molecules, based on the idea that complete T cell activation requires not only “signal 1” via the TCR, but also “signal 2” via co-stimulation (Figure 1) [105,106,107,108]. Different signaling domains grant the CAR cells specific attributes, such as enhanced cytotoxicity, prolonged survival, or stronger proliferation, and increase their overall potency, paving the way to employment against solid tumors [105,106,107,109,110,111,112,113,114,115,116,117,118,119,120].

Building on the success of co-stimulation in second-generation CARs, third-generation receptors have been assembled bearing not one but two or more co-stimulatory domains (Figure 1), expecting even enhanced cell activation, proliferation, and survival [121]. However, experiments showed that the effects of multiple signaling molecules are not contributing in an additive way, but are integrated in a far more complex manner [120]. Results from both preclinical experiments and clinical trials have been published, proving that some third-generation CARs can indeed be superior [73,121,122], while others fall behind [120,121,123] when directly compared to second-generation CARs. This mixed evidence demands for a better understanding of all influencing parameters to allow rational CAR design in the future [120,121,124,125].

Carrying the idea of the “three signals of T cell activation” one step further, CAR cells have been designed to additionally secrete certain cytokines, “signal 3” (Figure 1). These constructs, often revered to as TRUCKs, show the capability to enhance effector functions and durability of effector cells [120,121,126].

Besides the identity and number of employed signaling domains, the design chosen to integrate them into one CAR determines the properties of the receptor as a whole. Sequence and length of the linker and spacer region connecting the scFv domain to the signaling molecules have a strong impact on antigen recognition but also immunogenicity of the entire receptor [19,108,126,127]. The order of intracellular domains in the protein chain and their distance from the plasma membrane greatly impact the signaling competence [120,121,126,128,129,130].

Determining the ideal combination of signaling domains for a certain application is one of the ongoing challenges in the field of CAR therapy, especially when using alternative cell types. The following sections summarize properties and experimental data on the classical as well as uprising alternative signaling domains used in CARs over the last years.

### 3.1. Classical Signaling Domains

CD3ζ, CD28, and 4-1BB (i.e., CD137) today are the best studied signaling domains used in CAR constructs across different cell types. Years of pre-clinical testing with such constructs have led to the FDA approval of the first two CAR-T cell therapies in 2017 [9]: Kymriah and Yescarta, both directed against CD19 but containing different TM and signaling domains (CD8α(TM)-4-1BB/CD3ζ respectively IgG1(TM)-CD28/CD3ζ) [9], clearly making them the hallmarks of CAR research.

#### 3.1.1. CD3ζ

**Figure 2:**
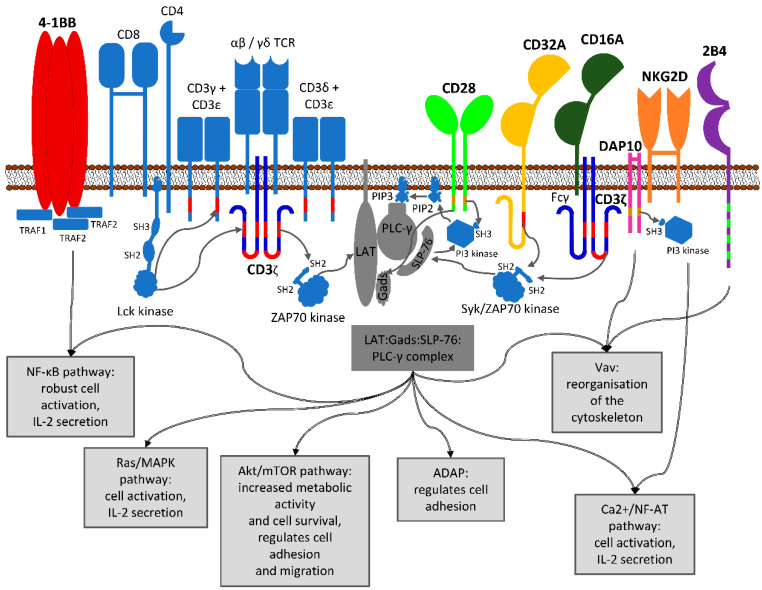
Summarizing representation of the normal roles of potential CAR signaling domains. Arrows indicate how the signaling pathways of different surface receptors are interconnected respectively to find resemblance in other cell types. Golden bars represent SH3 binding motifs, light green bars ITSMs, red bars ITAMs. More details on the individual proteins are discussed in the text.

Two CD3ζ chains, also called CD247, are part of the native T cell receptor complex. Together with one CD3γ and -δ chain and two CD3ε chains, they form an open barrel structure around a TCR α/β dimer respectively, a TCR γ/δ dimer, interacting via residues within the membrane interface [131,132] (Figure 2). In its intracellular part, each CD3ζ chain contains three tyrosine-rich sequences known as immunoreceptor tyrosine-based activation motifs (ITAMs) [133]. These are phosphorylated by membrane-localized Src-kinases upon TCR antigen binding, predominantly by Lyn, which associates with the MHC-binding co-receptors CD4 and CD8 to be brought in proximity of the TCR complex [134]. Phosphorylation renders ITAMs capable of binding the kinase ZAP70, leading to down-stream signaling via the MAPK, NF-κB, and NF-AT pathways in T cells. By promoting the transcription of corresponding genes, these pathways promote survival, proliferation, maturation, secretion of IL-2 and other cytokines, and cytolytic functions [135].

Although CD3ζ is only expressed on T and NK cells, signaling cascades involving ITAMs are present in all leucocytes, meaning that phosphorylation of CD3ζ can induce down-stream effects in different cell types. While the ITAM binding kinase ZAP70 is only expressed in T and NK cells, the closely related kinase Syk is present in all non-T leucocytes. In NK cells, neutrophils, macrophages, and mast cells, ITAM phosphorylation mimics Fc-receptor signaling and therefore triggers ADCC respectively to uptake of antibody-coated targets.

In first-generation CARs (Figure 1), the transmembrane domain of CD3ζ was used to connect the signaling domain to the extracellular scFv domain [8]. As the CD3ζ TM domain contains those residues essential for the association with the endogenous TCR complex, it was shown that these CARs have the ability to cluster with endogenous TCRs on the T cell surface [136]. In second- and third-generation CARs, it is very uncommon to use the CD3ζ TM domain, and most constructs rely on the CD28- or CD8α-TM domain [126,137].

#### 3.1.2. CD28

CD28 is a receptor expressed on T cells acting as a co-stimulatory signal supporting the TCR (Figure 2). Besides on T cells, CD28 is also expressed on eosinophils and neutrophils [138,139,140]. Its ligands are CD80 and CD86, present on antigen-presenting cells. As a co-stimulatory molecule, its absence or presence on antigen-presenting cells plays an important role in peripheral tolerance because naïve T cells can only become activated if the TCR and CD28 are simultaneously stimulated. When a T cell is interacting with an antigen-presenting cell, CD28 is a major driver in immunological synapse organization [141] by manipulating the actin cytoskeleton [142]. This rearrangement of the cytoskeleton also guarantees recruitment of binding partners in close proximity to the TCR complex and, for example, facilitates ITAM phosphorylation. In this manner, CD28 co-stimulation supports the MAPK, NF-κB, and NF-AT pathways, enhancing all effector functions stimulated by TCR signaling [143]. On a functional level, CD28 promotes the production of IL-2, -6, -10, and further interleukins, as well as cell cycle progression, survival, differentiation, and cytolytic functions [144].

A long list of studies employing CARs with a CD28 signaling domain reported potent and quick anti-tumor effector functions but found these to be rather short-lived and associated with limited cell persistence in vivo when compared to, e.g., the 4-1BB signaling domain [113,143,145,146]. CD28 co-stimulated CAR-T cells differentiate towards an effector-memory type which is characterized by secretion of IL-2 and IL-10, and an upregulated glycolytic metabolism to sustain their high energy demand [27,113,121,147,148,149,150,151,152]. In many second- and third-generation CAR constructs, the TM domain of CD28 is used as a connector of extra- and intra-cellular domains, which was associated with improved expression and presence of these CARs on the surface [153,154]. CD28 co-stimulation (potentially enhanced by the high expression levels) is thought to facilitate tonic CAR signaling [106,155] and thereby lead to Fas-dependent activation-induced cell death (AICD) in CAR-T cells [114]. This might also explain the observed limited cell persistence [113]. Clinical data mirrors preclinical findings: CD28 supports strong but short-lived anti-tumor efficacy [16,156,157]. Although CD28 is one of the most commonly employed signaling domains in CAR-T cells, it has been questioned whether CD28 can successfully enhance activation in CAR-NK cells [126], since NK cells lack naturally expressed CD28 [158,159].

#### 3.1.3. 4-1BB (CD137)

4-1BB is known as a co-stimulatory receptor that, together with others, is upregulated on the surface of activated T cells and promotes prolonged activation (Figure 2). Apart from activated T cells, it is expressed on a variety of lymphoid cells [160,161] as well as some non-hematopoietic tissues [162,163]. 4-1BB signaling in T cells enhances cell cycle progression and proliferation, cytokine secretion, cytolytic potential, and inhibits clonal deletion and AICD [164,165].

When employed as a CAR signaling domain (Figure 1), 4-1BB was shown to increase cytotoxicity above the level of first generation CARs and allow for more robust cell activation, as well as increase persistence in vivo [27,107,121,145,147,148,149,150,151,152,166]. By altering the cell’s metabolism towards enhanced respiratory capacity and fatty acid oxidation, 4-1BB co-stimulation promotes differentiation of CAR-T cells towards a central memory phenotype [27,113,121,147,148,149,150,151,152]. When compared to CD28, 4-1BB co-stimulated CARs showed slower onset of cytotoxicity, but longer durability and accumulation of CAR cells over time, which ultimately resulted in comparable efficacy but slower kinetics of the anti-tumor response [107].

Clinical trials similarly reported potent anti-tumor efficacy and very long persistence of 4-1BB-containing CAR-T cells in patients, with transgenic cells being detectable for up to several years [167,168]. Good in vivo persistence is a prerequisite to build up anti-tumor memory and long-term tumor control [107,167,168].

### 3.2. Non-Classical Extracellular and Signaling Domains

CAR constructs employing CD3ζ plus either CD28, 4-1BB, or both, have become state-of-the-art in laboratory work and clinical trials. To further develop CAR therapy regarding efficacy and safety, to direct it towards solid cancer types, and to bring new cellular vehicles into play, the optimization of the signaling capacity of CARs needs to be continued. Employing new signaling domains will reveal especially effective combinations among them and show specific potencies in different cell types. The following sections summarize properties and reported trials of a selection of proteins that are or might become emerging candidates for next generation CARs.

#### 3.2.1. NKG2D

NKG2D is a C-type lectin-like receptor expressed by NK, CD8^+^ α/β T, and γ/δ T cells (Figure 2) [27,169,170]. It is activated by induced self-proteins known as stress markers on aberrant, such as infected or malignant, cells [27,171,172,173,174]. The most prominent role of NKG2D is to drive NK cell-mediated cellular cytotoxicity against tumors [175]. In cytotoxic CD8^+^ T lymphocytes, the receptor delivers co-stimulation to TCR signaling [170]. Two NKG2D proteins form a dimer and each monomer complexes with one DAP10 dimer for down-stream signaling (Figure 2) [54,55,176,177], which is addressed in more detail below.

Being limited to just one antigen has ever since been the weak spot of scFv-based CARs, restricting their field of application to cancer types with known characterizing antigens, and bearing the risk of tumor antigen evasion. Several groups worked on a different approach on chimeric receptors, employing a complete NKG2D receptor molecule fused with the inverted intracellular domain of CD3ζ, called NKR2 (Figure 1) [178]. Instead of recognizing a specific antigen, this CAR’s extracellular domain binds stress markers just as the endogenous receptor does. The CAR intracellularly complexes with the adapter protein DAP10 for co-stimulatory signaling [55,176,177,179], while the CD3ζ domain drives cell activation and cytotoxicity [178]. Not being limited to one target antigen makes this CAR a promising tool against different cancer types and outplays the risk of tumor antigen evasion [178,180]. Preclinical data shows the NKR2 to be potent against a variety of hematologic and solid cancers [178,181,182,183]. NKR2 T cells have been translated to six phase 1/2 clinical trials (NCT02203825, NCT03018405, NCT03310008, NCT03370198, NCT03466320, and NCT03692429), employed against acute myeloid leukemia (AML), multiple myeloma (MM), melodysplastic syndrome, colorectal cancer, and colon cancer liver metastases.

A slightly different chimeric NKG2D receptor combines NKG2D with CD28 and CD3ζ signaling molecules [184]. Lentivirally transduced and mRNA-transfected T cells were tested against Ewing’s sarcoma family of tumors. Tumor cells were efficiently lysed by both CD8^+^ and CD4^+^ T cells, although the receptor disappeared from the surface of transiently transfected cells after a few hours [184].

While all of these CARs included the extracellular domain of the NKG2D receptor, Li et al. presented a CAR consisting of an anti-mesothelin (meso) scFv coupled to only the transmembrane domain of NKG2D plus 2B4 (see below) and CD3ζ signaling domains (Figure 1) [68]. The group pointed out that many previous studies of CAR-NK cells translated CAR constructs originally designed for the use in T cells to NK cells, although signaling domains result in different downstream effects dependent on the cell type. Adequate CAR constructs have to be chosen to unleash a vehicles full potential [68]. The group generated and screened nine CARs employing only domains with prominent roles in NK cells, chose the most promising candidate (anti-meso-NKG2D/2B4/CD3ζ), and went on to further characterizing it. NK cells derived from transduced induced pluripotent stem cell (iPSC) effectively inhibited cancer growth and prolonged survival in meso^+^ ovarian cancer-xenografted mice, outperforming iPSC NK cells expressing a pre-known CD28/4-1BB/CD3ζ CAR. When comparing the novel NKG2D/2B4/CD3ζ CAR-NK cells to T cells transduced with the CD28/4-1BB/CD3ζ CAR, both demonstrated very similar anti-tumor activity, fortifying the importance of vehicle-specific CAR design. In terms of persistence, the new NK-cell CAR was superior to its rival and resulted in less off-target toxicity. When tracking down the effector functions to the individual domains of the CAR via selective introduction of disruptive mutations, it became clear that the NKG2D and 2B4 domains both significantly contributed to activatory signaling, while CD3ζ only played a minor role in this construct [115].

#### 3.2.2. DAP10

An NKG2D dimeric receptor associates with two dimers of the adaptor protein called DNAX-activating protein of 10 kDa (DAP10) via their TM domains to form a hexameric complex (Figure 2) [54,55,176,177]. Upon antigen binding, the cytoplasmic domain of DAP10 becomes phosphorylated and recruits PI3K and a complex of GRB2 and VAV1 to trigger down-stream signaling cascades [54,55,176,177].

Comparing the cytokine profile of different CAR cells, secreted cytokines usually include pro-inflammatory IFNγ, TNF, IL-2, GM-CSF, IL-17, and IL-21, as well as anti-inflammatory IL-10 [152]. Unlike most other signaling domains, DAP10 was shown not to induce IL-10 secretion, but to strongly enhance T cell effector functions via pro-inflammatory cytokines [152,182,185,186,187,188,189,190,191,192,193]. Similar to CD28-bearing receptors, DAP10 promoted differentiation towards an effector memory phenotype in T cells, which was associated with enhanced persistence in vivo [27,121,147,148,149,150,151,152,182,185,186,187,188,189,190,191,192].

Several groups have performed studies on novel CAR constructs bearing a DAP10 signaling domain over the last years. Their results are summarized in the following. When elongating two second-generation CD28/4-1BB/CD3ζ CARs by a DAP10 domain, Zhao et al. found these receptors to have superior anti-tumor activity against GPC3^+^ lung cancer, respectively MSLN^+^ hepatocellular carcinoma and gastric cancer, prolonging the survival of xenograft-bearing mice [194,195].

In their proof-of-principle study, Duong et al. screened a variety of randomly assembled CARs for potent constructs. By this, they brought up a promising DAP10/CD3ζ/CD27 CAR and probed it in T cells versus ErbB2^+^ tumor cells, both in vitro and in a mouse model. They reported the new CAR to perform significantly better than an established CD28/CD3ζ CAR [196].

CARs based on the extracellular binding domain of PD-1 can be employed against a broad variety of cancer types that upregulate PD-1 ligands on their surface. When testing PD1-CD3ζ/DAP10 CAR-T cells in a mouse model of lymphoma, Lynch et al. saw significantly enhanced persistence compared to a CD28 co-stimulated CAR, leading to long-term tumor-free survival [192]. PD1-DAP10/CD3ζ CAR-T cells employed against syngeneic murine models of a variety of solid cancers (renal, pancreatic, liver, colon, breast, prostate, and bladder cancer, and melanoma) were also capable of inducing remission [193]. Both groups accredited the good performance to the preferential (IL-10-free) cytokine profile and memory phenotype differentiation induced by the DAP10 domain [192,193].

Two hurdles faced in CAR therapy that both result from powerful cell activation domains, are, on one side, on-target/off-tumor toxicity, and on the other side, tonic signaling, resulting in effector cell exhaustion. In an attempt to address both of these issues, Fisher et al. tested a “co-stimulation only” CAR (chimeric co-stimulation receptor, CCR), consisting of an anti-GD2 extracellular domain and DAP10 as the sole signaling domain (Figure 1), transduced in γ/δ T cells [197,198]. Because the DAP10 signal alone is not sufficient for activation, these T cells depend on simultaneous activation of their native γ/δ TCR [197]. In vitro, the CCR T cells efficiently lysed neuroblastoma cells but spared GD2^+^ cells lacking γ/δ TCR ligands [197]. CCR T cells also differentiated between healthy and malignant myeloid target cells [198], making chimeric co-stimulation receptors a very safe potential alternative to established CARs.

While the NKR2 receptor described in the chapter above consisted only of NKG2D and a CD3ζ signaling domain, Chang et al. designed a CAR additionally bearing the DAP10 domain (Figure 1) in both retrovirally transduced and mRNA-electroporated NK cells [180]. The CAR cells showed potent anti-tumor activity in a mouse model of osteosarcoma which is unresponsive to native NK cells, but no comparison to other receptors was performed [180].

Two trials have been conducted employing NK cells, respectively cytokine-induced killer cells transduced with anti-CD19 first- and second-generation CARs bearing a DAP10 signaling domain (Figure 1) [166,199]. Unlike the ones listed above, these trials yielded dampening results: although DAP10 signaling is a strong trigger of cytotoxicity in native NK cells [200,201], those CARs induced weaker anti-tumor activity in direct comparison to receptors containing CD3ζ alone, respectively 4-1BB/CD3ζ or CD28/CD3ζ [166,202]. These findings stand in clear contrast to others listed above. A better understanding of all factors influencing the potency of these particular CAR constructs is needed to draw reliable conclusions.

#### 3.2.3. Fcγ-receptors

By binding to the Fc part of antibodies, Fc-receptors induce immune cell effector functions towards antibody-bound targets (Figure 2) [203,204,205]. They drive phagocytosis of antibody-opsonized extracellular pathogens by macrophages, dendritic cells, and neutrophils, as well as ADCC by NK cells, eosinophils, basophils, and mast cells via exocytosis of cytotoxic mediators [203,204,205]. Besides these activatory functions, some Fc-receptors also pursue regulatory roles, facilitate antigen uptake and presentation, and allow placental transport of antibodies [206]. Receptors in this family differ in their specificity and affinity for certain antibody classes and subclasses, and are selectively expressed by different leucocytes [204]. Due to their strong activatory properties in many cell types, Fc-receptors are an interesting group of proteins to be employed in CARs.

##### FcγRIII (CD16)

The best-studied Fc-receptor is the type III Fcγ receptor (FcγRIII), also known as CD16 (Figure 2), which binds IgG1 and 3 with moderate affinity [203,207,208,209]. Two subtypes of this receptor, A and B, sharing 96% sequence identity [210], are characteristically expressed on certain cell types: type A plays the most prominent role by guiding NK cells towards antibody-opsonized cells and inducing ADCC—leading to the release of perforin and granzymes from granules. It also stimulates cytokine release in macrophages [207,208]. Type B initiates exocytosis of granules, mainly against microbes in eosinophils, macrophages, neutrophils, and mast cells [207,208]. To initiate down-stream signaling, FcγRIIIA associates with a dimer of the Fcγ adaptor protein, which is closely related to CD3ζ, or a CD3ζ dimer, or a heterodimer of the two (Figure 2), depending on the native expression of these proteins is the respective cell [209]. Phosphorylation of ITAMs on these adapter proteins initiates the next steps in the signaling cascade [207,208].

Different strategies have been tested to develop CARs not limited to just one tumor antigen. Universal CARs (uniCARs) do not have a covalently linked scFv but can reversibly complex with antibodies. One way to implement this is to generate Fcγ chimeric receptors (Fcγ CRs) by coupling intracellular co-stimulatory plus signaling domains to an extracellular CD16 domain, which then binds to the Fc part of antibodies [211,212]. Fcγ CRs can synergize with antibodies naturally present in the patient and be redirected to specific target antigens by administration of therapeutic monoclonal antibodies (mAbs) [211,212]. In contrast to scFv-based CARs, this allows to employ Fcγ CR cells for a broad variety of malignancies for which therapeutic mAbs are available, and to fine-tune the therapy for the individual patient via frequency and dosage [211,212]. The usage of a cocktail of different mAbs reduces the risk of antigen evasion and allows to react to uprising tumor cells with an altered phenotype [211,212]. In terms of safety, the therapy can be adjusted to control toxicity and, if required, quickly be interrupted by ending mAb administration [211,212].

Fcγ CRs have been pre-clinically tested by different groups [213,214,215,216,217,218,219]. Summarizing these studies, CD16A or a high-affinity variant (V158) was either coupled directly or via a CD8α linker and TM domain to a signaling tail, consisting of CD3ζ, 4-1BB/CD3ζ, or FcεRIγ (Figure 1). These constructs have been retrovirally transduced or transfected [214,215] into human T cells, murine MD45 T cells, human peripheral blood NK cells, or NK-92 cells. These cells have been employed in combination with a variety of mAbs (anti-CD19, anti-CD20 (Rituximab), anti-HER2 (Trastuzumab), anti-GD2, anti-Panc1, anti-EGFR (Cetuximab, Panitumumab), or anti-CSPG4) against different cancer cells (Daudi, Raji, and adult T cell lymphoma, neuroblastoma, osteosarcoma, pancreatic cancer, colorectal cancer, and A375 melanoma cells) in vitro, as well as against lymphoma-xenografted mice [213,214,215,216,217,218,219]. Fcγ CR cells showed promising anti-tumor activity in all these studies [213,214,215,216,217,218,219], and via the attached signaling domains, activated the NF-AT pathway [213]. Fcγ CR T cells expressing the 4-1BB/CD3ζ signaling domains sustained especially potent proliferation, and stronger activation and cytotoxicity than with CD3ζ or FcεRIγ [214]. Fcγ CR T cells preferentially differentiated towards the favorable effector memory phenotype [213]. In direct comparison, Fcγ CR NK cells achieved comparable ADCC, but showed less proliferation than Fcγ CR T cells, causing decreased persistence and reduced long-term tumor control [213].

Fcγ CRs were the first implementation of uniCARs to reach the clinical phase [212]. A total of three phase 1 and one phase 1/2 trials have been registered (clinicaltrials.gov: NCT02315118, NCT02776813, NCT03266692, and NCT03189836), three employing CD16(V158)-4-1BB/CD3ζ autologous T cells in combination with Rituximab against refractory or relapsed lymphoma, respectively with BCMA against relapsed or refractory multiple myeloma. The fourth trial also targets refractory or relapsed lymphoma using Rituximab and an unknown construct in T cells.

##### FcγRIIA (CD32A)

The type II Fcγ receptor subtype A (FcyRIIA) (Figure 2) binds all subtypes of IgG with moderate affinity. It is expressed on macrophages and neutrophils, where it facilitates phagocytosis, and, on eosinophils, where it triggers degranulation. Besides that, the receptor is found on platelets and Langerhans cells. Unlike FcγRIII, FcγRIIA does not need to associate with Fcγ or CD3ζ for signaling. It possesses an Fcγ-like intracellular domain containing one ITAM that can be phosphorylated by Src kinases (Figure 2).

Based on the emergence of Fcγ CRs using a CD16 extracellular domain, Caratelli et al. designed a CD32A131R (low-affinity variant) chimeric receptor with a CD8α TM and a CD28/CD3ζ signaling domain to be retrovirally transduced into peripheral blood T cells [220]. Contrary to CD16, CD32A has moderate affinity also for IgG2, which is important in the treatment of epidermal growth factor receptor (EGFR)-positive tumors: beside Cetuximab (IgG1), Panitumumab (IgG2) is used as a therapeutic antibody. Directly comparing CD32A CR- to CD16 CR-T cells in combination with both those antibodies in vitro showed superior lytic capacity of the former construct, benefiting from both mAbs directing ADCC, while CD16 can only bind one of them [220].

The group went on translating their approach to KRAS-mutated colorectal cancer (CRC), which evades NK cell-mediated ADCC when treated only with Cetuximab and Panitumumab [219]. The previously presented CD32A131R CD8αTM/CD28/CD3ζ CR was compared to CD32131H, CD16158F, and CD16158V CRs. In this setting, CD32 CR-T cells failed to produce pro-inflammatory cytokines or exhibited cytotoxicity when co-cultured with opsonized KRAS-mutated HCT116 cells, although successfully binding the mAbs, while the CD16158V CR was found potent both in vitro and in vivo [219].

#### 3.2.4. 2B4 (CD244)

The signaling lymphocyte activation molecule (SLAM)-related receptor 2B4 (Figure 2) is a non-MHC-restricted immune regulatory trans-membrane receptor expressed predominantly on NK cells and some T cells (NKT, γ/δ T, and a portion of CD8^+^ T cells), but also on eosinophils, basophils, mast cells, monocytes, and dendritic cells [221,222]. Together with other SLAM family proteins, it contributes to the formation of the immunological synapse in NK and T cells, on one hand modulating cellular cytotoxicity, mediating a potent (co-)stimulatory signal, or on the other hand, driving cells into exhaustion; depending on multiple, not yet entirely understood factors [221,222,223,224,225,226]. Binding of its ligand, CD48, triggers phosphorylation of immunoreceptor tyrosine-based switch motifs (ITSMs) in the cytoplasmic tail of 2B4 (Figure 2) [115]. Of the four ITSMs present, only the first and second are associated with activatory signaling in NK cells [226]. When driving an activatory response, 2B4 is thought to act mainly as a co-stimulatory receptor crosslinking with, e.g., CD226 or NKG2D, and activating PLC-γ2, triggering Ca^2+^ influx, stimulating NK cell degranulation, proliferation, IL-2 secretion, and IFNγ release [63,222,226].

It has been shown that 2B4 expression is altered under specific pathologic conditions and might contribute to immune suppression in the TME [222]. Inhibitory signaling of 2B4 was predominantly found in chronic infection or in tumor-associated NK and T cells, seemingly being dependent on surface density of 2B4 and SLAM-associated protein (SAP), a binding partner of CD48: a high ratio of 2B4 to SAP correlates with inhibitory signaling, a low ratio with activatory signaling [222]. As these findings are mainly derived from NK cell studies, little is known about the role of 2B4 and its divergently expressed binding partners in other cell types [222].

2B4 has been studied as a signaling domain in CAR-NK cells by Altvater et al.: a CD19-specific scFv coupled to CD3ζ or 2B4 alone, 2B4/CD3ζ, or 4-1BB/CD3ζ signaling domains were tested against the leukemic cell line REH in vitro [225]. 2B4 was truncated to remove the latter two ITSMs which might drive inhibitory signaling [225]. 2B4 alone yielded a CAR that showed very weak activation, being less potent than a CD3ζ CAR, but combining 2B4 and CD3ζ performed similarly well to 4-1BB/CD3ζ [225,226]. Building on these findings, anti-GD2-2B4/CD3ζ CAR-NK cells were successfully employed against neuroblastoma cells in vitro [225]. To survey the effects of 2B4 signaling on T cells, the anti-CD19, respectively anti-GD2-2B4/CD3ζ CARs were transduced into peripheral blood T cells, yielding results comparable to NK cells, as presented above [226]. Proliferation of these T cells predominantly gave rise to the effector memory subset, which is associated with sustained strong anti-tumor capacity. The tumor-protective Treg subset was not expanded, supporting the role of 2B4 as a co-stimulatory receptor in T cells [226].

As described above in more detail, Li et al. designed a novel CAR specifically for the use in NK cells [68]. Anti-meso-NKG2D/2B4/CD3ζ CAR iPSC NK cells outperformed NK cells expressing an established CD28/4-1BB/CD3ζ CAR. It also had an edge over T cells expressing this classical CAR, when tested in meso+ ovarian cancer xenografted mice. NKG2D and 2B4 were both shown to contribute to the CAR’s performance [68].

In another study aiming to prove the importance of vehicle-specific CAR design, two anti-CD5 CARs with a 4-1BB/CD3ζ (a T cell-associated) or a 2B4/CD3ζ (rather NK cell-specific) intracellular tail were tested [227]. Both were transduced into NK cells and employed in a CD5^+^ T cell acute lymphoblastic leukemia (T-ALL) mouse model, where they both exhibited specific cytotoxicity and prolonged survival of the mice. The 2B4/CD3ζ CAR displayed greater lytic capacity but also elevated toxicity against non-malignant T cells [227].

In summary, the above shows that many alternative extracellular domains and intracellular signaling modules besides the classical scFv, CD3ζ, CD28, or 4-1BB domains are already tested for functionality in CARs. It is pivotal, however, to find ideal combinations between cellular vessels and CARs with different signaling moieties.

## Figures and Tables

**Table 1:** List of clinical trials employing non-classical/alternative chimeric antigen receptor (CAR) vehicles or combinations listed on www.clinicaltrials.gov.

Cell Type	Notes	Treatment	Cancer Types	Locations	NCT Number ^1^
γ/δ T cells	Not yet recruiting	anti-CD19-CAR γ/δ T cells	Different B-cell lymphomas	Beijing, China	NCT02656147
	Recruiting; observational only	determining number and viability of patients’ γ/δ T cells in preparation for a CAR trial	Acute myeloid leukemia.	Sutton, United Kingdom	NCT03885076
	Not yet recruiting	NKG2D-CAR γ/δ T cells	Different solid tumors	Johor Bahru, Malaysia	NCT04107142
NKT cells	Withdrawn, replaced by NCT03294954	iC9-anti-GD2-CD28/OX40/CD3ζ CAR NKT (GINAKIT) cells	Neuroblastoma	Houston, Texas, USA	NCT02439788
	Recruiting; new CAR construct	anti-GD2-CD28/CD3ζ-IL15 CAR NKT (GINAKIT2) cells	Neuroblastoma	Houston, Texas, USA	NCT03294954
	Not yet recruiting	anti-CD19-CD28/CD3ζ-IL15 CAR NKT (ANCHOR) cells	Different B-cell lymphomas	Houston, Texas, USA	NCT03774654
Combining CAR-T cells and DC vaccination	Recruiting	CAR-T cells, engineered immune effector (EIE) CTLs, DCvac(no details available)	Neurofibromatosis	Shenzhen, Guangdong, China	NCT04085159
	Recruiting	Different CAR-T cells, Eps8 or WT1 DCvac	Relapsed/refractory Leukemia, myelodysplastic syndrome	Guangzhou, Guangdong, China	NCT03291444

^1^ NCT number: CLINICALTRIALS.GOV IDENTIFIER.

**Table 2:** List of clinical trials employing CAR-NK cells listed on www.clinicaltrials.gov.

Notes	Treatment	Cancer Types	Locations	NCT Number ^1^
Completed	anti-CD19-4-1BB/CD3ζ CAR-NK cells	Acute lymphoblastic leukemia	Memphis, Tennessee, USA	NCT00995137
Suspended	anti-CD19-CD3ζ/4-1BB CAR-NK cells	B-cell acute lymphoblastic leukemia	Singapore	NCT01974479
Unknown status	anti-CD7-CD3ζ/CD28/4-1BB CAR NK-92 cells	Acute myeloid leukemia and 8 more hematologic cancers	Suzhou, Jiangsu, China	NCT02742727
Unknown status	anti-MUC1 CAR-NK cells	Hepatocellular carcinoma and 6 more solid cancers	Suzhou, Jiangsu, China	NCT02839954
Unknown status	anti-CD19-TCRζ/CD28/4-1BB CAR-NK cells	Acute lymphocytic leukemia and 5 more hematologic cancers	Suzhou, Jiangsu, China	NCT02892695
Unknown status	anti-CD33-CD3ζ/CD28/4-1BB CAR-NK cells	Acute myelogenous leukemia and 4 more hematologic cancers	Suzhou, Jiangsu, China	NCT02944162
Recruiting; results published	anti-CD19-CD28/CD3ζ/iCasp9/ IL-15 CAR-NK cells	Different B-cell lymphomas	Houston, Texas, USA	NCT03056339
Recruiting	NKG2D-CAR-NK cells	Different solid tumors	Guangzhou, Guangdong, China	NCT03415100
Not yet recruiting	anti-PSMA CAR-NK cells	Castration-resistant prostate cancer	Beijing, China	NCT03692663
Not yet recruiting	anti-CD19/CD22 CAR-NK cells	Refractory B-cell lymphoma	Beijing, China	NCT03824964
Not yet recruiting	anti-CD22 CAR-NK Cells	Refractory B-cell lymphoma	Beijing, China	NCT03692767
Not yet recruiting	anti-meso CAR-NK Cells	Epithelial ovarian cancer	Beijing, China	NCT03692637
Not yet recruiting	anti-CD19 CAR-NK Cells	Refractory B-cell lymphoma	Beijing, China	NCT03690310
Recruiting	anti-BCMA CAR-NK-92 cells	Multiple myeloma	Wuxi, Jiangsu, China	NCT03940833
Recruiting	anti-ROBO1 CAR-NK cells	Pancreatic cancer	Shanghai, China	NCT03941457
Recruiting	anti-ROBO1 CAR-NK cells	Different solid tumors	Suzhou, Jiangsu, China	NCT03940820
Withdrawn; lack of funding	anti-CD19-CD28/CD3ζ/iCasp9/IL-15 CAR-NK cells	Different B-cell lymphomas	Houston, Texas, USA	NCT03579927

^1^ NCT number: CLINICALTRIALS.GOV IDENTIFIER.

## Abbreviations

CAR	Chimeric antigen receptor
TCR	T cell receptor
MHC	Major histocompatibility complex
APC	Antigen-presenting cell
T_E_	Effector T cell
T_M_	Memory T cell
T_CM_	Central memory T cell
T_EM_	Effector memory T cell
ALL	Acute lymphoblastic leukemia
TME	Tumor microenvironment
CRS	Cytokine release syndrome
GvHD	Graft versus host disease
HSP	Heat shock protein
PPR	Pattern recognition receptor
IEL	Intraepithelial lymphocyte
ADCC	Antibody-dependent cellular cytotoxicity
NCR	Natural cytotoxicity receptor
KIR	Killer cell immunoglobulin-like receptor
CLL	Chronic lymphocytic leukemia
HSC	Hematopoietic stem cell
EGFR	Epidermal growth factor receptor
TAM	Tumor-associated macrophage
BMDM	Bone marrow-derived macrophage
CAR-P	Chimeric antigen receptor for phagocytosis
CARMA	CAR macrophage
TIR	Toll interleukin receptor
DC	Dendritic cell
DCvac	DC vaccination
Eps8	EGFR pathway substrate 8
EV	Extracellular vesicle
scFv	Single-chain variable fragment
TM	Transmembrane
ITAM	Immunoreceptor tyrosine-based activation motif
AICD	Activation-induced cell death
AML	Acute myeloid leukemia
MM	Multiple myeloma
meso	Mesothelin
iPSC	Induced pluripotent stem cell
DAP10	DNAX-activating protein of 10 kDa
CCR	Chimeric co-stimulatory receptor
uniCAR	Universal CAR
Fcγ CR	Fcγ chimeric receptor
mAb	Monoclonal antibody
EGFR	Epidermal growth factor receptor
CRC	Colorectal cancer
SLAM	Signaling lymphocyte activation molecule
ITSM	Immunoreceptor tyrosine-based switch motif
SAP	SLAM-associated protein
T-ALL	T cell acute lymphoblastic leukemia
